# dbPPT: a comprehensive database of protein phosphorylation in plants

**DOI:** 10.1093/database/bau121

**Published:** 2014-12-20

**Authors:** Han Cheng, Wankun Deng, Yongbo Wang, Jian Ren, Zexian Liu, Yu Xue

**Affiliations:** ^1^Department of Biomedical Engineering, College of Life Science and Technology, Huazhong University of Science and Technology, Wuhan, Hubei 430074, China and ^2^State Key Laboratory of Biocontrol, School of Life Sciences, Sun Yat-sen University, Guangzhou, Guangdong 510275, China

## Abstract

As one of the most important protein post-translational modifications, the reversible phosphorylation is critical for plants in regulating a variety of biological processes such as cellular metabolism, signal transduction and responses to environmental stress. Numerous efforts especially large-scale phosphoproteome profiling studies have been contributed to dissect the phosphorylation signaling in various plants, while a large number of phosphorylation events were identified. To provide an integrated data resource for further investigations, here we present a comprehensive database of dbPPT (database of Phosphorylation site in PlanTs, at http://dbppt.biocuckoo.org), which contains experimentally identified phosphorylation sites in proteins from plants. The phosphorylation sites in dbPPT were manually curated from the literatures, whereas datasets in other public databases were also integrated. In total, there were 82 175 phosphorylation sites in 31 012 proteins from 20 plant organisms in dbPPT, presenting a larger quantity of phosphorylation sites and a higher coverage of plant species in comparison with other databases. The proportions of residue types including serine, threonine and tyrosine were 77.99, 17.81 and 4.20%, respectively. All the phosphoproteins and phosphorylation sites in the database were critically annotated. Since the phosphorylation signaling in plants attracted great attention recently, such a comprehensive resource of plant protein phosphorylation can be useful for the research community.

**Database URL:** http://dbppt.biocuckoo.org

## Introduction

As one of the most ubiquitous post-translational modifications (PTMs), protein phosphorylation is critical for almost all types of cellular processes such as cell cycle and signaling transduction (1, 2). Through transferring a phosphate group from ATP to the hydroxyl group of a residue, in most cases, serine, threonine and tyrosine (3), phosphorylation could alter the structural and/or functional state of the protein (4). Phosphorylation was reversibly catalyzed by kinases and phosphatase (3), and then contributed to the dynamic cellular signaling in organisms. In plants, phosphorylation plays important roles in a variety of critical processes such as regulation of photosystem (5), metabolism (6). For example, early in 1980s, Bennett *et al*. (7) found that a membrane-bound protein kinase could regulate excitation energy transfer through phosphorylation of surface-exposed segments of the light-harvesting pigment–protein complex in the photosystem. Bachmann *et al*. (8) identified S543 as the major phosphorylation site which could regulate nitrate metabolism through control the activity of leaf nitrate reductase in spinach. Recently, phosphorylation was found to be heavily involved in the plant circadian system through various aspects including circadian oscillator function, circadian entrainment, circadian output pathways and circadian regulations of cellular processes such as ABA signaling (9). Accumulated studies also showed that phosphorylation was highly implicated in the response of various stresses (10). Taken together, understanding of phosphorylation signaling is critical to reveal the physiological and pathological processes in plants.

Recently, rapid progress in phosphopeptide enrichment methods and mass spectrum (MS)-based identification techniques greatly advanced large-scale detection of phosphorylation events in plants (11). Early in 2004, Heintz *et al*. (12) published a protocol for identification of phosphorylation in moss *Physcomitrella patens*, with a detection of 253 distinct phosphopeptides. However, although the phosphoproteomics techniques in yeast and mammals developed quickly, phosphoproteome analysis in plants is still challenging (13). In 2008, Sugiyama *et al*. (14) identified over 2000 of phosphotyrosine sites in *Arabidopsis* with the extent of phosphotyrosines, demonstrating the great advancement of phosphoproteomics in plants (15). Quantitative techniques were also introduced (16, 17), while recently a number of studies employed quantitative phosphoproteomic to study the phosphorylation signaling dynamics in plants (18–20). With the accumulation of studies on phosphorylation in plants, a large number of phosphoproteins and sites were identified, while data maintenance and sharing became increasingly challenging.

To deposit the precious phosphorylation information for easy retrieving, a handful of studies have been contributed. Although databases such as PhosphoSitePlus (21), Phospho.ELM (22), dbPTM (23) and SysPTM (24) provided comprehensive information of phosphorylation sites, these databases were mainly focused on yeast and animals, while data for plant is limited. Meanwhile, a dozen of databases focused on plants were developed, such as PlantsP (25), PhosPhAt (26) and P^3^DB (27). However, there were limited phosphorylation data or lower coverage of species in most of these databases. For example, PlantsP contains only 4969 phosphoproteins (25), while PhosPhAt and MPPD are exclusively developed for *Arabidopsis* and *Medicago*, respectively (26, 28). Thus, a more comprehensive database is needed for further studies of phosphorylation in plants.

In this study, we provided a large collection of 82 175 phosphorylation sites in 31 012 phosphoproteins that were manually curated from literatures. The proportions for phosphorylated serines, threonines and tyrosines were 77.99, 17.81 and 4.20%, respectively. The phosphoproteins were from 20 plant species, whereas 29 058 phosphorylation sites in 9003 phosphoproteins were from *Arabidopsis*. Most phosphorylation sites were mapped to the sequences from the UniProt database. All the phosphoproteins were well annotated with various descriptions and the source references for phosphorylation identifications were also provided. We further constructed the database of dbPPT (database of Phospho-sites in PlanTs) to provide user-friendly online services for data retrieval and access. The keyword-based search and blast search services were implemented. The browse service was also provided to query information for a specific organism of interest. As an application of the dbPPT database, the most significant sequence motifs for phosphorylation in *Arabidopsis* were analyzed. Taken together, dbPPT serves as the most comprehensive data resource for phosphorylation in plants, and would be helpful for the research community

## Database construction and content

To construct the most comprehensive database for phosphorylation in plants, we integrated data from multiple different sources including PubMed, P^3^DB and PhosPhAt. The literature database of PubMed (http://www.ncbi.nlm.nih.gov/pubmed) was searched to retrieve published plant phosphorylation-related studies through various keywords combinations such as ‘plant(s) phosphoproteomic’, ‘plant(s) phosphoproteome’ and more than 20 plant species names combined with ‘phosphoproteomic’ or ‘phosphoproteome’ like ‘*Arabidopsis thaliana* phosphoproteomic’, ‘*Arabidopsis thaliana* phosphoproteome’. To avoid missing data, additional keywords like ‘plant(s) large-scale phosphorylation’, ‘plant(s) MS phosphorylation’ and other related keywords were also employed to search PubMed. The retrieved literatures were manually checked to collect the experimentally identified phosphorylation substrates and sites in plants. The collected sites were cross checked with other databases such as P^3^DB and PhosPhAt.

Since there might be differences among protein sequences from different versions or sources, we employed the UniProt (Release 2014_06) database (29) as the source of benchmark sequences. For most species, their sequences could be found in UniProt. However, the sequences for a number of species such as *Brassica napus* could not be fully mapped to UniProt entries. Therefore, sequence information from other public databases such as Ensembl (Release 75) (30) and NCBI Protein (31) were employed as the benchmark to map the collected phosphorylation sites. A full list of the databases used in this study was provided in Supplementary Table S1. To provide further information for the curated phosphoproteins, the annotations were also retrieved from the UniProt and other public databases. To ensure the high quality of dbPPT and allow the user to query further information about the identified phosphorylation sites, the primary references were also provided.

**Figure 1.**
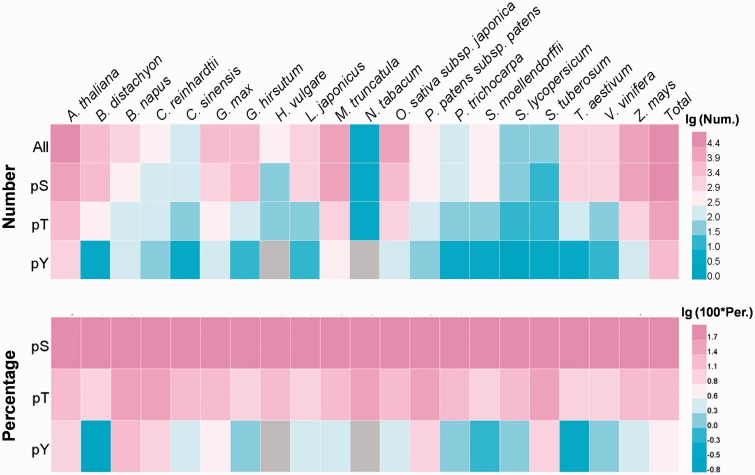
The heatmap for the distribution of site numbers and percentages for phosphoserine (pS), phosphothreonine (pT) and phosphotyrosine (pY) in different species.

In total, 82 175 identified phosphorylation sites in 31 012 phosphoproteins were provided in dbPPT, while the numbers (and proportions) of phosphoserine, phosphothreonine and phosphotyrosine were 64 089 (77.99%), 14 632 (17.81%) and 3454 (4.20%), respectively (Supplementary Table S2). Obviously, most of the identified phosphorylation sites were phosphoserines, while the number of phosphotyrosine was limited. However, with the advancement of phosphoproteomics techniques, more tyrosine phosphorylation will be identified (32). As the model organism for plants, *Arabidopsis* attracted special attentions from the research community as 29 058 phosphorylation sites in 9003 proteins were identified. Other plants especially the crops also have considerable number of identified phosphorylation events. For *Zea mays, Medicago truncatula* and *Oryza sativa* subsp. *Japonica*, there were 13 580, 14 836 and 8603 identified phosphorylation sites, respectively. The heatmaps for the data summarization of the numbers and proportions of phosphorylation sites were presented in Figure 1, while the details were shown in Supplementary Table S2. All the phosphorylation sites and sequences of proteins in the dbPPT database can be downloaded at http://dbppt.biocuckoo.org/download.php.

## Database usage

**Figure 2.**
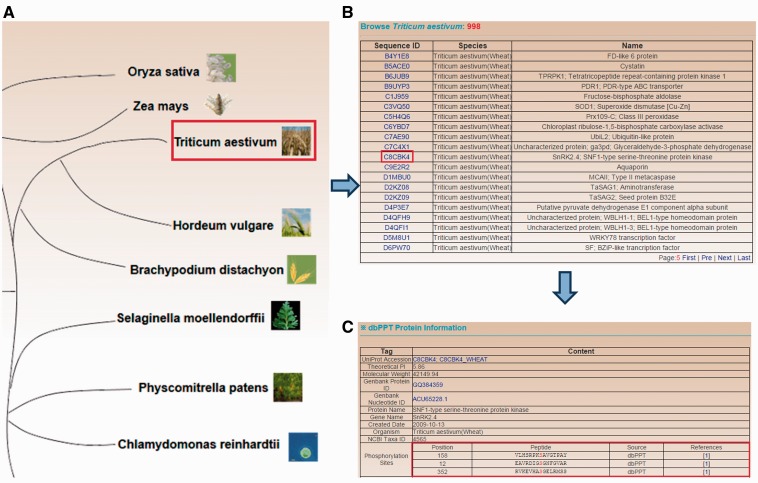
The browse option developed for browsing by plant species in the dbPPT database. (A) The 20 plant species which contain identified phosphorylation sites. (B) The list of identified phosphoprotein in *T. aestivum*. (C) The detailed information of *T. aestivum* SNF1-type serine–threonine protein kinase.

To provide convenient services for the research community, browse and search functions with various options are developed in a user-friendly manner for the dbPPT database. The browse function is developed for organism-based querying among the 20 plant species. Using the important crop wheat as an example, the workflow for browsing is shown in Figure 2. The plant species are presented in an evolutionary tree on the ‘BROWSE’ page with images and organism names, while user could click the image or organism name for one species to browse the phosphoproteins (Figure 2A). If ‘*Triticum aestivum’* is selected, then all the identified phosphoproteins of wheat are listed (Figure 2B). The dbPPT entry, sequence ID (the ID in the database from which the sequence of the protein is retrieved, for example, UniProt Accesion) and the protein names (including gene name, gene synonyms, protein name and protein synonyms) are shown for browsing. User could click the dbPPT entry for the protein of interest to view the details of the phosphoprotein (Figure 2B). General information for the protein such as protein ID and gene ID are shown in this page (Figure 2C). The phosphorylation sites are presented with position number, peptide with 7 amino acids upstream and downstream of the modified residue, the data source of the site and the link to the primary reference (Figure 2C). The ‘dbPPT’ in the ‘Source’ column means the site is collected in this study, while other such as ‘P^3^DB’ represents that the site is cross checked with the P^3^DB database. Annotations such as gene ontology and Interpro are also provided for further understanding of the phosphoprotein.

**Figure 3.**
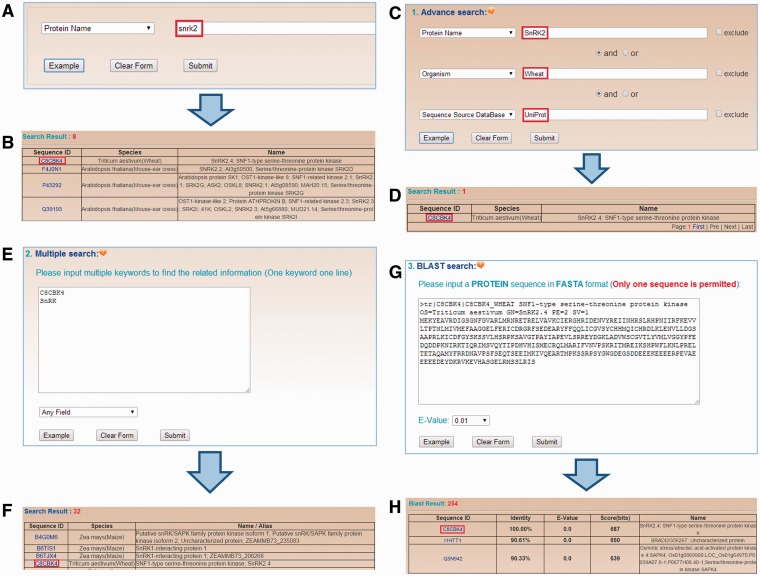
The search options in the dbPPT database. (A) dbPPT can be queried with a simple keyword. (B) The results of simple search for ‘snrk2’ in the area ‘Protein Name’. (C) Advanced search allows users to perform a complex search using multiple keywords in several areas and three operators including ‘and’, ‘or’ and ‘exclude’. (D) Advanced search result of ‘SnRK2’ in wheat while its full-length sequence was retrieved from UniProt. (E) Multiple search allows user to search multiple keywords in one query event. (F) The results of searching ‘C8CBK4’ and ‘SnRK’ in any field. (G) BLAST search was implemented for search similar phosphoproteins with FASTA format protein sequence. (H) BLAST search for SNF1-type serine–threonine protein kinase in wheat.

To provide convenient querying services, four search options are implemented in dbPPT. In the home page of dbPPT, a simple search option is provided, which allows user to perform keyword-based search in specific area (Figure 3A). An example with SNF1-type serine–threonine protein kinase SnRK2.4 is shown by searching the keyword ‘snrk2’ in the ‘Protein Name’ area. After submitting the query, the results will be shown in a tabular format with dbPPT ID, Sequence ID and protein/gene names/synonyms (Figure 3B). Furthermore, three additional search options including advanced search, multiple search, BLAST search are also implemented and available in the ‘SEARCH’ page of dbPPT. In advanced search option, three keywords can be used to perform a complex search in three areas through combination with three operators of ‘and’, ‘or’ and ‘exclude’ (Figure 3C), while the results are shown in the format as the simple search option (Figure 3D). In multiple search option, several keywords such as a list of gene names can be used to search one area (Figure 3E), all the proteins hits any one of these keywords will be presented in the results (Figure 3F). In addition, the BLAST search option is designed to search phosphoproteins in dbPPT with a protein sequence in the FASTA format. User could submit a protein sequence to run a homologous search through NCBI BLAST packages (33) with a specified *E*-Value threshold (Figure 3G). Search results of BLAST search is presented in a tabular format with dbPPT ID, sequence similarity evaluations including identity, E-value and score, sequence ID and protein/gene name/synonyms (Figure 3H). Furthermore, all the phosphoproteins in the dbPPT database could be accessed directly through the link of ‘http://dbppt.biocuckoo.org/getTable. php?seqID=accession’, while the ‘accession’ is the sequence accession such as UniProt accession.

### Conclusion and future direction

As one of the most important PTMs, protein phosphorylation was involved in almost all aspects of biology processes (1, 2). Phosphorylation in yeast and animals was extensively studied in recent years while tens of thousands of phosphorylation sites were identified (21–24). Although the advancement for understanding of phosphorylation in plants is limited by comparison with yeast and animals, recently more and more studies were contributed to this area to dissect the important roles of phosphorylation in the physiological and pathological processes in plants (11). Besides the requirement for maintaining the phosphorylation data which was in explosive increase, a comprehensive data resource was the foundation of systematic analyses of phosphorylation signaling. Although a number of studies have been contributed (25–28), more efforts are needed.

**Figure 4.**
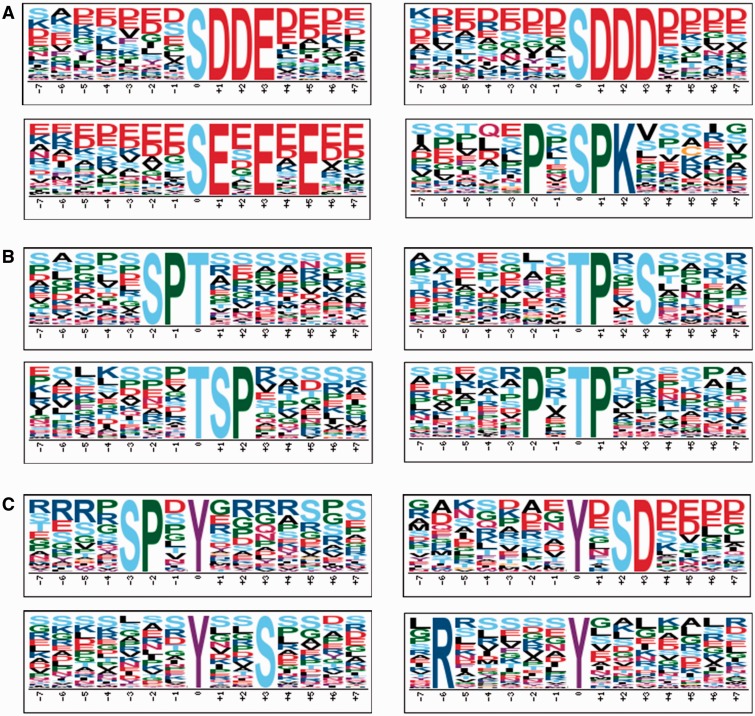
The top four most representative motifs discovered by Motif-x for phosphoserine, phosphothreonine and phosphotyrosine in *Arabidopsis*. (A) Motifs for phosphoserine. (B) Motifs for phosphothreonine. (C) Motifs for phosphotyrosine. The position 0 represents the modified residues.

In this study, we constructed the dbPPT database with 82 175 phosphorylation sites in 31 012 proteins, which could be employed to perform systematic studies. Since *Arabidopsis* was the well-studied organism in plants, we analyzed the sequence motifs for phosphorylation. The motifs for phosphoserine, phosphothreonine and phosphotyrosine were calculated with Motif-x (Supplemental Tables S3–S5) (34), while the top four motifs with the highest motif scores were presented in Figure 4. For phosphoserine, the top four most representative motifs were pS-D-D-E, pS-D-D-D, pS-E-*X*-E-*X*-E and P-*X*-pS-P-K (pS, pT and pY represent the phosphoserine, phosphothreonine and phosphotyrosine, respectively. *X* represents any amino acid) (Figure 4A). The top four motifs with the highest scores for phosphothreonine were S-P-pT, pT-P-*X*-S, pT-S-P and P-*X*-pT-P (Figure 4B). Previously, it was proposed that [ST]-P motif might be recognized by MAPKs and CDKs (22), which indicated that MAPKs and CDKs might be the primary kinases for the identified phosphoserines and phosphothreonines. Acidic motifs such as pS-D-*X*-[DE], pS-E-*X*-E and pS-[DE] were potential substrates of CKII (35, 36), while other motifs such as R-S-*X*-pS, L-*X*-R-*X-X*-pS and R-*X-X*-pS were associated with CPKs and CaMKs activity (36, 37). The top four most representative motifs for phosphotyrosine were calculated as S-P-*X*-pY, pY-*X*-S-D, pY-*X-X*-S and R-*X-X-X-X-X*-pY, while the potential kinases for these motifs needed to be dissected. In general, most of the motifs such as [ST]-P, pS-D-*X*-[DE], R-*X-X*-pS were conserved between plants and mammals, which indicated that their corresponding kinases played crucial roles in both mammals and plants.

Besides the results of motif analyses, which might be helpful for prediction of phosphorylation sites in plants, actually, PhosphAt database provided prediction service for *Arabidopsis* (26). To evaluate the coverage of positive hits on experimentally identified phosphorylation sites for the prediction function of PhosphAt, the 94 284 predicted phosphorylation sites from 25 840 substrates in PhosphAt were downloaded for computation. To avoid redundancy in the two datasets, the phosphopeptides with six amino acids upstream and six amino acids downstream around the modified residue were employed for comparison, which resulted in 75 343 and 25 640 non-redundant tridecapeptides for PhosphAt and dbPPT, respectively. From comparison, it was observed that there were 3966 overlapping phosphopeptides between the two datasets, which indicated 15.47% of experimentally identified phosphorylation sites could be predicted as positive hits.

With the development of phosphoproteome techniques, more efforts will be contributed to this area. The systematic methods to dissect phosphorylation signaling in yeast and animals such as network-based analyses could be employed in plants (38), while the kinase and phosphatase information in plants could also be integrated to reveal the reversible regulation of phosphorylation (39). Furthermore, other PTMs such as acetylation and ubiquitination could also be employed to dissect the PTMs signaling in plants (40, 41). Taken together, here we built the dbPPT database with the manually curated phosphorylation events, which will be a useful resource for both computational and experimental scientists. The dbPPT database will be routinely updated to follow research progresses of protein phosphorylation studies in plants.

## Supplementary Data

Supplementary data are available at *Database* Online.

Supplementary Data
