# Supplementary material for: dbPPT: a comprehensive database of protein phosphorylation in plants
Source: Database (Oxford). 2014 Dec 20;2014:bau121. doi: 10.1093/database/bau121 (PMC4273206; doi:10.1093/database/bau121)
Supplement: Supplementary Data [file supp_2014_bau121_index.html]

dbPPT: a comprehensive database of protein phosphorylation in plants — Supplementary Data 

# dbPPT: a comprehensive database of protein phosphorylation in plants

## Supplementary Data

files

**Files in this Data Supplement:**

- Supplementary Data - docx file
