# Supplementary material for: dbPPT: a comprehensive database of protein phosphorylation in plants
Source: Database (Oxford). 2014 Dec 20;2014:bau121. doi: 10.1093/database/bau121 (PMC4273206; doi:10.1093/database/bau121)
Supplement: Supplementary Data [file supp_bau121_supplementary_data_20141119_R_lzx.docx]

**Supplementary Table S1** - A full list of the databases from which benchmark sequences were obtained.

| **Database** | **Link** |
| --- | --- |
| Uniprot | http://www.uniprot.org/ |
| NCBI | http://www.ncbi.nlm.nih.gov/protein/ |
| DFCI | http://compbio.dfci.harvard.edu/tgi/plant.html |
| phytozome | http://www.phytozome.net/ |
| LegumeIP | http://plantgrn.noble.org/LegumeIP/ |
| Miyakogusa.jp | http://www.kazusa.or.jp/lotus/ |
| The Medicago truncatula genome project Mt3.0 | http://www.jcvi.org/medicago/ |
| Rice Genome Annotation Project | http://rice.plantbiology.msu.edu/ |
| Ensembl | http://plants.ensembl.org/index.html |
| CGP | http://cgp.genomics.org.cn/page/species/index.jsp |
| TAIR | http://arabidopsis.org/ |

**Supplementary Table S2** - Data summarization for the collected identified phosphoserine, phosphothreonine and phosphotyrosine in 20 plant species.

| **Species** | **Protein** | **Site** | **Serine (Num./Per.)** | **Threonine (Num./Per.)** | **Tyrosine (Num./Per.)** |
| --- | --- | --- | --- | --- | --- |
| *Arabidopsis thaliana* | 9,003 | 29,058 | 20,545 (70.70%) | 6,517 (22.43%) | 1,996 (6.87%) |
| *Brachypodium distachyon* | 1,680 | 3,239 | 2,861 (88.33%) | 373 (11.52%) | 5 (0.15%) |
| *Brassica napus* | 325 | 818 | 484 (59.17%) | 218 (26.65%) | 116 (14.18%) |
| *Chlamydomonas reinhardtii* | 218 | 527 | 292 (55.41%) | 181 (34.35%) | 54 (10.25%) |
| *Citrus sinensis* | 111 | 182 | 133 (73.08%) | 42 (23.08%) | 7 (3.85%) |
| *Glycine max* | 1,436 | 2,718 | 2,181 (80.24%) | 396 (14.57%) | 141 (5.19%) |
| *Gossypium hirsutum* | 1,474 | 2,789 | 2,491 (89.32%) | 270 (9.68%) | 28 (1.00%) |
| *Hordeum vulgare* | 345 | 523 | 457 (87.38%) | 66 (12.62%) | 0 (0.00%) |
| *Lotus japonicus* | 489 | 904 | 789 (87.28%) | 95 (10.51%) | 20 (2.21%) |
| *Medicago truncatula* | 4,204 | 14,836 | 12,211 (82.31%) | 2,283 (15.39%) | 342 (2.31%) |
| *Nicotiana tabacum* | 8 | 9 | 5 (55.56%) | 4 (44.44%) | 0 (0.00%) |
| *Oryza sativa subsp. japonica* | 3,746 | 8,603 | 7,048 (81.92%) | 1,243 (14.45%) | 312 (3.63%) |
| *Physcomitrella patens subsp. patens* | 374 | 747 | 428 (57.30%) | 230 (30.79%) | 89 (11.91%) |
| *Populus trichocarpa* | 200 | 297 | 243 (81.82%) | 50 (16.84%) | 4 (1.35%) |
| *Selaginella moellendorffii* | 716 | 730 | 634 (86.85%) | 91 (12.47%) | 5 (0.68%) |
| *Solanum lycopersicum* | 43 | 60 | 48 (80.00%) | 11 (18.33%) | 1 (1.67%) |
| *Solanum tuberosum* | 21 | 33 | 17 (51.52%) | 12 (36.36%) | 4 (12.12%) |
| *Triticum aestivum* | 998 | 1,499 | 1,363 (90.93%) | 130 (8.67%) | 6 (0.40%) |
| *Vitis vinifera* | 694 | 1,023 | 919 (89.83%) | 93 (9.09%) | 11 (1.08%) |
| *Zea mays* | 4,927 | 13,580 | 10,940 (80.56%) | 2,327 (17.14%) | 313 (2.30%) |
| Total | 31,012 | 82,175 | 64,089 (77.99%) | 14,632 (17.81%) | 3,454 (4.20%) |

**Supplementary Table S3** - Motifs discovered by Motif-x around phosphoserines.

| No. | **Motif** | **Motif Score** | **Foreground Matches** | **Foreground Size** | **Background Matches** | **Background Size** | **Fold Increase** |
| --- | --- | --- | --- | --- | --- | --- | --- |
| 1 | ....RS.SP...... | 39.84 | 72 | 17161 | 498 | 1013104 | 8.54 |
| 2 | ....RS.S....... | 32 | 409 | 17089 | 6199 | 1012606 | 3.91 |
| 3 | .....P.SPK..... | 43.84 | 52 | 16680 | 191 | 1006407 | 16.43 |
| 4 | .......SP.R.... | 32 | 317 | 16628 | 2711 | 1006216 | 7.08 |
| 5 | ......RSPS..... | 41.62 | 47 | 16311 | 308 | 1003505 | 9.39 |
| 6 | .......SPS..... | 32 | 310 | 16264 | 6282 | 1003197 | 3.04 |
| 7 | .....S.SP...... | 32 | 287 | 15954 | 5200 | 996915 | 3.45 |
| 8 | ......RS.S..... | 32 | 299 | 15667 | 6133 | 991715 | 3.09 |
| 9 | ...S...SPR..... | 40.55 | 50 | 15368 | 321 | 985582 | 9.99 |
| 10 | .......S...SPR. | 38.67 | 44 | 15318 | 412 | 985261 | 6.87 |
| 11 | ...S...SP...... | 32 | 230 | 15274 | 4127 | 984849 | 3.59 |
| 12 | .......SP..S... | 32 | 256 | 15044 | 3806 | 980722 | 4.38 |
| 13 | .....SPS....... | 31.48 | 206 | 14788 | 5825 | 976916 | 2.34 |
| 14 | .R..S..S....... | 32 | 231 | 14582 | 6025 | 971091 | 2.55 |
| 15 | .......S.SP.... | 32 | 225 | 14351 | 5557 | 965066 | 2.72 |
| 16 | .....S.SE.E.... | 36.02 | 46 | 14126 | 650 | 959509 | 4.81 |
| 17 | ..L.R..S..S.... | 39.56 | 54 | 14080 | 621 | 958859 | 5.92 |
| 18 | .......SDDE.... | 48 | 119 | 14026 | 540 | 958238 | 15.06 |
| 19 | ......DSD.E.... | 38.43 | 51 | 13907 | 330 | 957698 | 10.64 |
| 20 | ......PSSP..... | 38.96 | 56 | 13856 | 502 | 957368 | 7.71 |
| 21 | ....KS.SF...... | 35.56 | 26 | 13800 | 270 | 956866 | 6.68 |
| 22 | .......SD.E.E.. | 42.74 | 51 | 13774 | 390 | 956596 | 9.08 |
| 23 | .......SD.EE... | 41.58 | 39 | 13723 | 301 | 956206 | 9.03 |
| 24 | .......SE.E.E.. | 45.2 | 64 | 13684 | 593 | 955905 | 7.54 |
| 25 | ....R..SP...... | 32 | 129 | 13620 | 1579 | 955312 | 5.73 |
| 26 | .......SPRS.... | 40.03 | 31 | 13491 | 169 | 953733 | 12.97 |
| 27 | .......SSP..... | 32 | 324 | 13460 | 6710 | 953564 | 3.42 |
| 28 | ...R.S.S....... | 25.56 | 142 | 13136 | 4819 | 946854 | 2.12 |
| 29 | .......S.DE.... | 32 | 160 | 12994 | 3621 | 942035 | 3.2 |
| 30 | .......SDSD.... | 34.49 | 40 | 12834 | 590 | 938414 | 4.96 |
| 31 | .......SDDD.... | 48 | 82 | 12794 | 506 | 937824 | 11.88 |
| 32 | ....R..SG...... | 30.18 | 118 | 12712 | 3021 | 937318 | 2.88 |
| 33 | K......SP...... | 32 | 94 | 12594 | 1678 | 934297 | 4.16 |
| 34 | .......SDED.... | 40.85 | 43 | 12500 | 360 | 932619 | 8.91 |
| 35 | ......GSP...... | 32 | 86 | 12457 | 1431 | 932259 | 4.5 |
| 36 | .......S.SE.... | 26.84 | 155 | 12371 | 5384 | 930828 | 2.17 |
| 37 | .......SP.S.... | 32 | 124 | 12216 | 2593 | 925444 | 3.62 |
| 38 | .......S.GE.... | 24.7 | 111 | 12092 | 3434 | 922851 | 2.47 |
| 39 | .......SDEE.... | 31.82 | 31 | 11981 | 361 | 919417 | 6.59 |
| 40 | .......SP..E... | 31.11 | 72 | 11950 | 1335 | 919056 | 4.15 |
| 41 | ......DS.E..... | 24.94 | 103 | 11878 | 2932 | 917721 | 2.71 |
| 42 | ......GSG...... | 23.7 | 147 | 11775 | 5382 | 914789 | 2.12 |
| 43 | .......S.DDE... | 38.94 | 37 | 11628 | 318 | 909407 | 9.1 |
| 44 | ..L.R..S....... | 30.84 | 180 | 11591 | 4058 | 909089 | 3.48 |
| 45 | .......S...SP.. | 32 | 183 | 11411 | 5019 | 905031 | 2.89 |
| 46 | ..R..S.S....... | 24.75 | 127 | 11228 | 4682 | 900012 | 2.17 |
| 47 | K...R..S....... | 23.06 | 76 | 11101 | 2282 | 895330 | 2.69 |
| 48 | .......SP...E.. | 29.21 | 60 | 11025 | 1176 | 893048 | 4.13 |
| 49 | R......S.G..... | 23.69 | 89 | 10965 | 2907 | 891872 | 2.49 |
| 50 | .......S.DD.... | 29.09 | 116 | 10876 | 3056 | 888965 | 3.1 |
| 51 | .K.....SP...... | 22.01 | 40 | 10760 | 1035 | 885909 | 3.18 |
| 52 | .......SPE..... | 22.62 | 54 | 10720 | 1471 | 884874 | 3.03 |
| 53 | .......SE.D.... | 24.29 | 96 | 10666 | 3065 | 883403 | 2.59 |
| 54 | K......S....... | 16 | 843 | 10570 | 51436 | 880338 | 1.37 |
| 55 | .......S.E..... | 16 | 742 | 9727 | 45413 | 828902 | 1.39 |
| 56 | .......SD.D.... | 29.34 | 89 | 8985 | 2205 | 783489 | 3.52 |
| 57 | .......S.S..... | 16 | 1195 | 8896 | 82987 | 781284 | 1.26 |
| 58 | .......SPK..... | 25.62 | 75 | 7701 | 978 | 698297 | 6.95 |
| 59 | .......S.G..... | 16 | 730 | 7626 | 48789 | 697319 | 1.37 |
| 60 | ....R..S.D..... | 25.16 | 58 | 6896 | 1522 | 648530 | 3.58 |
| 61 | .......SPR..... | 25.45 | 74 | 6838 | 1055 | 647008 | 6.64 |
| 62 | ....R..S....... | 16 | 441 | 6764 | 24975 | 645953 | 1.69 |
| 63 | .......SP.K.... | 22.74 | 49 | 6323 | 748 | 620978 | 6.43 |
| 64 | .E.....SP...... | 22.6 | 45 | 6274 | 719 | 620230 | 6.19 |
| 65 | .......S.D..D.. | 23.39 | 62 | 6229 | 2031 | 619511 | 3.04 |
| 66 | .....S.S....... | 16 | 777 | 6167 | 57862 | 617480 | 1.34 |
| 67 | .......SP...... | 16 | 304 | 5390 | 12190 | 559618 | 2.59 |
| 68 | .......SD.E.... | 22.05 | 53 | 5086 | 1538 | 547428 | 3.71 |
| 69 | .K.....S....... | 12.74 | 426 | 5033 | 32184 | 545890 | 1.44 |
| 70 | ......SS....... | 12.9 | 675 | 4607 | 57081 | 513706 | 1.32 |
| 71 | ......GS.R..... | 18.55 | 55 | 3932 | 2128 | 456625 | 3 |
| 72 | ......DS....... | 10.62 | 319 | 3877 | 25573 | 454497 | 1.46 |
| 73 | ....K..S....... | 10.43 | 331 | 3558 | 27654 | 428924 | 1.44 |
| 74 | ...S...S....... | 9.38 | 440 | 3227 | 40945 | 401270 | 1.34 |
| 75 | .......S.K..... | 8.08 | 289 | 2787 | 26672 | 360325 | 1.4 |
| 76 | .......S...D... | 7.63 | 199 | 2498 | 17775 | 333653 | 1.5 |
| 77 | .......S...K... | 8.55 | 222 | 2299 | 20379 | 315878 | 1.5 |
| 78 | .......S.R..... | 7.43 | 206 | 2077 | 19939 | 295499 | 1.47 |
| 79 | ...K...S....... | 8.12 | 184 | 1871 | 17584 | 275560 | 1.54 |
| 80 | .....R.S....... | 6.8 | 142 | 1687 | 13857 | 257976 | 1.57 |
| 81 | ..K....S....... | 6.75 | 135 | 1545 | 13479 | 244119 | 1.58 |

**Supplementary Table S4** - Motifs discovered by Motif-x around phosphothreonines.

| **No.** | **Motif** | **Motif Score** | **Foreground Matches** | **Foreground Size** | **Background Matches** | **Background Size** | **Fold Increase** |
| --- | --- | --- | --- | --- | --- | --- | --- |
| 1 | [.....P.TP......](http://motif-x.med.harvard.edu/cgi-bin/jobres.pl?jobid=20141117-21120-36892268#.....P.TP......) | 29.89 | 133 | 5623 | 2521 | 571083 | 5.36 |
| 2 | [.....SPT.......](http://motif-x.med.harvard.edu/cgi-bin/jobres.pl?jobid=20141117-21120-36892268#.....SPT.......) | 32 | 120 | 5490 | 3042 | 568562 | 4.09 |
| 3 | [......RT.S.....](http://motif-x.med.harvard.edu/cgi-bin/jobres.pl?jobid=20141117-21120-36892268#......RT.S.....) | 26.75 | 83 | 5370 | 2778 | 565520 | 3.15 |
| 4 | [.......TP.S....](http://motif-x.med.harvard.edu/cgi-bin/jobres.pl?jobid=20141117-21120-36892268#.......TP.S....) | 32 | 93 | 5287 | 2748 | 562742 | 3.6 |
| 5 | [.......TPT.....](http://motif-x.med.harvard.edu/cgi-bin/jobres.pl?jobid=20141117-21120-36892268#.......TPT.....) | 22.66 | 66 | 5194 | 1499 | 559994 | 4.75 |
| 6 | [.......TSP.....](http://motif-x.med.harvard.edu/cgi-bin/jobres.pl?jobid=20141117-21120-36892268#.......TSP.....) | 32 | 101 | 5128 | 2945 | 558495 | 3.74 |
| 7 | [.......TP......](http://motif-x.med.harvard.edu/cgi-bin/jobres.pl?jobid=20141117-21120-36892268#.......TP......) | 16 | 467 | 5027 | 22130 | 555550 | 2.33 |
| 8 | [.......T.SP....](http://motif-x.med.harvard.edu/cgi-bin/jobres.pl?jobid=20141117-21120-36892268#.......T.SP....) | 26.36 | 72 | 4560 | 2530 | 533420 | 3.33 |
| 9 | [.......T..SP...](http://motif-x.med.harvard.edu/cgi-bin/jobres.pl?jobid=20141117-21120-36892268#.......T..SP...) | 22.01 | 66 | 4488 | 2716 | 530890 | 2.87 |
| 10 | [.K.....T.......](http://motif-x.med.harvard.edu/cgi-bin/jobres.pl?jobid=20141117-21120-36892268#.K.....T.......) | 13.83 | 403 | 4422 | 32603 | 528174 | 1.48 |
| 11 | [......ST.......](http://motif-x.med.harvard.edu/cgi-bin/jobres.pl?jobid=20141117-21120-36892268#......ST.......) | 12.17 | 498 | 4019 | 44680 | 495571 | 1.37 |
| 12 | [.......TS..E...](http://motif-x.med.harvard.edu/cgi-bin/jobres.pl?jobid=20141117-21120-36892268#.......TS..E...) | 16.04 | 55 | 3521 | 2577 | 450891 | 2.73 |
| 13 | [.......T..S....](http://motif-x.med.harvard.edu/cgi-bin/jobres.pl?jobid=20141117-21120-36892268#.......T..S....) | 9.55 | 415 | 3466 | 39580 | 448314 | 1.36 |
| 14 | [.......TD.E....](http://motif-x.med.harvard.edu/cgi-bin/jobres.pl?jobid=20141117-21120-36892268#.......TD.E....) | 19.82 | 52 | 3051 | 1933 | 408734 | 3.6 |
| 15 | [R..S...T.......](http://motif-x.med.harvard.edu/cgi-bin/jobres.pl?jobid=20141117-21120-36892268#R..S...T.......) | 15.14 | 45 | 2999 | 1945 | 406801 | 3.14 |
| 16 | [.......T.E.....](http://motif-x.med.harvard.edu/cgi-bin/jobres.pl?jobid=20141117-21120-36892268#.......T.E.....) | 7.56 | 282 | 2954 | 27805 | 404856 | 1.39 |
| 17 | [..D....T.......](http://motif-x.med.harvard.edu/cgi-bin/jobres.pl?jobid=20141117-21120-36892268#..D....T.......) | 7.49 | 208 | 2672 | 19900 | 377051 | 1.47 |
| 18 | [...K...T.......](http://motif-x.med.harvard.edu/cgi-bin/jobres.pl?jobid=20141117-21120-36892268#...K...T.......) | 7.44 | 221 | 2464 | 22071 | 357151 | 1.45 |
| 19 | [.R.....T.......](http://motif-x.med.harvard.edu/cgi-bin/jobres.pl?jobid=20141117-21120-36892268#.R.....T.......) | 7.41 | 186 | 2243 | 18456 | 335080 | 1.51 |
| 20 | [....K..T.......](http://motif-x.med.harvard.edu/cgi-bin/jobres.pl?jobid=20141117-21120-36892268#....K..T.......) | 7.27 | 183 | 2057 | 18748 | 316624 | 1.5 |
| 21 | [.......T.....K.](http://motif-x.med.harvard.edu/cgi-bin/jobres.pl?jobid=20141117-21120-36892268#.......T.....K.) | 6.26 | 165 | 1874 | 17711 | 297876 | 1.48 |

**Supplementary Table S5** - Motifs discovered by Motif-x around phosphotyrosines.

| **No.** | **Motif** | **Motif Score** | **Foreground Matches** | **Foreground Size** | **Background Matches** | **Background Size** | **Fold Increase** |
| --- | --- | --- | --- | --- | --- | --- | --- |
| 1 | [.......Y..S....](http://motif-x.med.harvard.edu/cgi-bin/jobres.pl?jobid=20141117-20634-08973240#.......Y..S....) | 10.41 | 231 | 1789 | 26502 | 318025 | 1.55 |
| 2 | [....SP.Y.......](http://motif-x.med.harvard.edu/cgi-bin/jobres.pl?jobid=20141117-20634-08973240#....SP.Y.......) | 17.9 | 33 | 1558 | 1271 | 291523 | 4.86 |
| 3 | [.......Y.SD....](http://motif-x.med.harvard.edu/cgi-bin/jobres.pl?jobid=20141117-20634-08973240#.......Y.SD....) | 14.76 | 34 | 1525 | 1516 | 290252 | 4.27 |
| 4 | [.R.....Y.......](http://motif-x.med.harvard.edu/cgi-bin/jobres.pl?jobid=20141117-20634-08973240#.R.....Y.......) | 7.58 | 131 | 1491 | 15366 | 288736 | 1.65 |
| 5 | [......KY.......](http://motif-x.med.harvard.edu/cgi-bin/jobres.pl?jobid=20141117-20634-08973240#......KY.......) | 6.79 | 125 | 1360 | 15553 | 273370 | 1.62 |
| 6 | [.......YS......](http://motif-x.med.harvard.edu/cgi-bin/jobres.pl?jobid=20141117-20634-08973240#.......YS......) | 6.58 | 151 | 1235 | 20744 | 257817 | 1.52 |
| 7 | [.......Y.....K.](http://motif-x.med.harvard.edu/cgi-bin/jobres.pl?jobid=20141117-20634-08973240#.......Y.....K.) | 6.71 | 112 | 1084 | 14809 | 237073 | 1.65 |
